# Supplementary material for: Identification of photoperiod-induced specific miRNAs in the adrenal glands of Sunite sheep (Ovis aries)
Source: Front Vet Sci. 2022 Jul 22;9:888207. doi: 10.3389/fvets.2022.888207 (PMC9354845; doi:10.3389/fvets.2022.888207)
Supplement: Supplementary file 1 [file Data_Sheet_1.ZIP › Supplementary file/miRDeep2 file.zip!/miRDeep2 file/Know_Structure/SPLP42A1/oar-mir-376e.pdf]

miRBase precursor : oar-mir-376e
Total read count : 1032
oar-miR-376e-5p read count : 200
oar-miR-376e-3p read count : 632
oar-miR-376b-5p read count : 200
remaining reads : 0

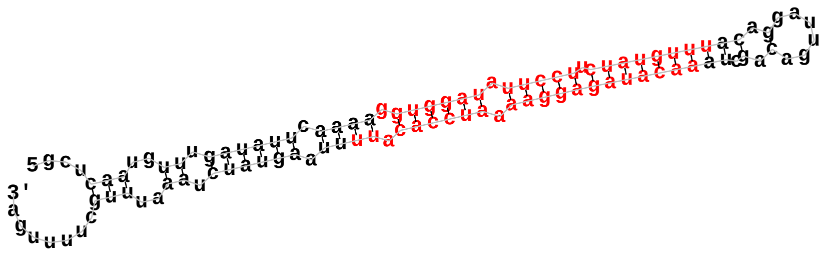

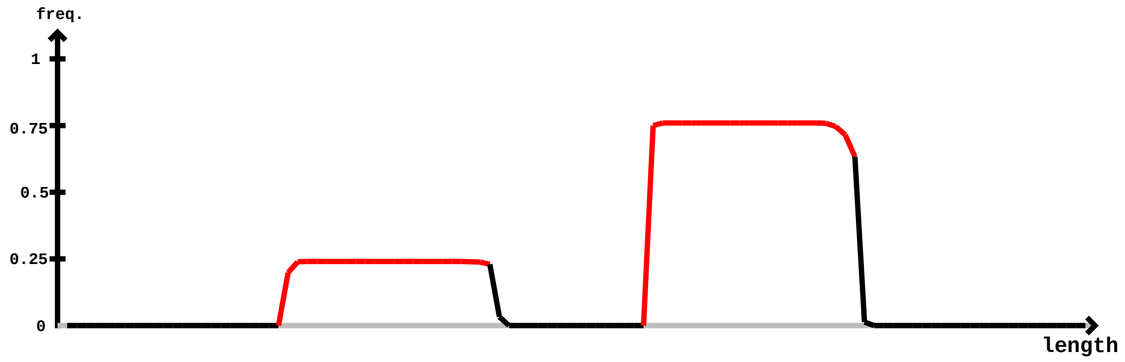


```
                                                                oar-miR-376e-3p
                        oar-miR-376b-5p
                        oar-miR-376e-5p
5'- gcucaauguuugauauucaaaagguggauauuccuucuauguuuacaggauugacagcuaaacauagaggaaaauccacauuuuaaguaucuaaauuugcuuuuga  -3'   exp
    ((.....((((((((((.((((.((((((.(((((.((((((((((.(......).).)))))))))))))).)))))).)))).)))))).))))..)).....   reads   mm   sample
    ......................gguggauauuccuucuaug.................................................................   1       0    seq
    ......................gguggauauuccuucuauguu...............................................................   5       0    seq
    ......................gguggauauuccuucuauguuu..............................................................   135     0    seq
    ......................gguggauauuccuucuauguuua.............................................................   24      0    seq
    .......................guggauauuccuucuaugu................................................................   1       0    seq
    .......................guggauauuccuucuauguu...............................................................   2       0    seq
    .......................guggauauuccuucuauguuu..............................................................   29      0    seq
    .......................guggauauuccuucuauguuua.............................................................   2       0    seq
    ........................uggauauuccuucuauguuua.............................................................   1       0    seq
    ............................................................aacauagaggaaaaucca............................   1       0    seq
    ............................................................aacauagaggaaaauccac...........................   10      0    seq
    ............................................................aacauagaggaaaauccaca..........................   26      0    seq
    ............................................................aacauagaggaaaauccacau.........................   67      0    seq
    ............................................................aacauagaggaaaauccacauu........................   511     0    seq
    ............................................................aacauagaggaaaauccacauuu.......................   9       0    seq
    .............................................................acauagaggaaaauccacau.........................   1       0    seq
    .............................................................acauagaggaaaauccacauu........................   5       0    seq
    .............................................................acauagaggaaaauccacauuu.......................   2       0    seq
```
